# LncRNA TMEM220-AS1 suppresses hepatocellular carcinoma cell proliferation and invasion by regulating the TMEM220/β-catenin axis

**DOI:** 10.7150/jca.63351

**Published:** 2021-09-24

**Authors:** Yang Liu, Runkun Liu, Junjun Zhao, Zhi Zeng, Zhan Shi, Qiliang Lu, Jinhui Guo, Lijie Li, Yingmin Yao, Xin Liu, Qiuran Xu

**Affiliations:** 1 The Medical College of Qingdao University, Qingdao, 266071, China.; 2 The Key Laboratory of Tumor Molecular Diagnosis and Individualized Medicine of Zhejiang Province, Zhejiang Provincial People's Hospital, Affiliated People's Hospital, Hangzhou Medical College, Hangzhou 310014, China.; 3 Department of Hepatobiliary Surgery, The First Affiliated Hospital of Xi'an Jiaotong University, Xi'an 710061, China.; 4 Graduate Department, Bengbu Medical College, Bengbu 233030, China.; 5 The Second Clinical Medical College, Zhejiang Chinese Medical University, Hangzhou 310053, China.; 6 Department of Obstetrics and Gynaecology, Affiliated Zhejiang Hospital, Zhejiang University School of Medicine, Hangzhou 310013, China.

**Keywords:** Hepatocellular carcinoma, TMEM220-AS1, TMEM220, β-catenin, Tumor progression

## Abstract

Long non-coding RNAs (lncRNAs) are critical drivers and suppressors of human hepatocellular carcinoma (HCC). The downregulation of transmembrane protein 220 antisense RNA 1 (TMEM220-AS1) is correlated with poor prognosis in HCC. Nevertheless, the role of TMEM220-AS1 in HCC and the underlying mechanism remains unclear. In this study, TMEM220-AS1 levels were markedly reduced in HCC tissues compared with noncancerous tissues. TMEM220-AS1 downregulation was confirmed in HCC cell lines. TMEM220-AS1 expression was associated with tumor stage, venous infiltration, tumor size, and survival of HCC patients. TMEM220-AS1 overexpression suppressed the migration, invasion, and proliferation of HCC cells. Interestingly, ectopic expression of TMEM220-AS1 increased TMEM220 levels in HCC cells. Decreased TMEM220 levels were observed in HCC tissues and cell lines. TMEM220 expression was positively correlated with TMEM220-AS1 levels in HCC tissue samples and TMEM220 downregulation was significantly correlated with reduced patient survival. TMEM220 overexpression suppressed HCC cell proliferation and mobility. TMEM220 knockdown eliminated the suppressive effect of TMEM220-AS1 in HCCLM3 cells. Mechanistically, TMEM220 overexpression reduced the nuclear accumulation of β-catenin and decreased MYC, Cyclin D1, and Snail1 mRNA levels in HCCLM3 cells. BIO, a GSK3β inhibitor, eliminated TMEM220-induced Wnt/β-catenin pathway inactivation and inhibited HCC cell proliferation and mobility. In conclusion, TMEM220-AS1 and TMEM220 were expressed at low levels in HCC patients. TMEM220-AS1 inhibited the malignant behavior of HCC cells by enhancing TMEM220 expression and subsequently inactivating the Wnt/β-catenin pathway.

## Introduction

The most recent data from the International Agency for Research on Cancer (IARC) indicates that primary liver cancer is the fifth most common cancer and the second leading cause of cancer-related deaths in China 1. Hepatocellular carcinoma (HCC) accounts for approximately 90% of all liver cancers. The five-year survival rate for patients with HCC who undergo hepatectomy is less than 20% in China 2. Thus, multiple studies have focused on investigating the mechanisms underlying hepatocarcinogenesis and developing new drugs targeting HCC.

RNA transcripts longer than 200 nucleotides which lack significant protein-coding ability are called long noncoding RNAs (lncRNAs) 3. An increasing number of studies have demonstrated the critical functions of lncRNAs in the occurrence and progression of human HCC 4-6. We have previously investigated the impact of lncRNAs, including MCM3AP-AS1, PICSAR, LINC01123, and A1BG-AS1, in HCC tumor progression 7-10. MCM3AP-AS1 is highly expressed in HCC, and its overexpression predicts poor prognosis in HCC 7. MCM3AP-AS1 competitively binds miR-194-5p to enhance FOXA1 expression and facilitates HCC cell growth 7. LINC01123 is a tumor-promoting factor that exerts an oncogenic effect in HCC cell proliferation, migration, and invasiveness by modulating the miR-34a-5p/TUFT1 axis 9. The upregulation of PICSAR and its association with poor prognosis were reported in our previous study 8. PICSAR contributes to HCC progression by modulating the miR-588/EIF6 axis and activating the PI3K/AKT/mTOR pathway 8. A1BG-AS1 functions as a tumor suppressor and inhibits the proliferation, migration, and invasion of tumor cells by targeting miR-216-5p in HCC 10. A recent study reported the downregulation of transmembrane protein 220 antisense RNA 1 (TMEM220-AS1) and its copy number variation in HCC 11. Moreover, low TMEM220-AS1 expression predicts poor prognosis in HCC 11. Nevertheless, the role of TMEM220-AS1 in HCC and the underlying mechanism of action remains unclear. TMEM220-AS1 is an antisense lncRNA of TMEM220. High TMEM220 methylation and its downregulation have been detected in gastric cancer tissues 12. However, it is unknown whether TMEM220 acts as a functional target of TMEM220-AS1.

Here, we analyzed TMEM220-AS1 and TMEM220 expression in HCC and adjacent non-tumor tissues and determined their prognostic significance. We investigated the effects of TMEM220-AS1 and TMEM220 on cell proliferation, invasion, and migration. The potential pathways regulated by TMEM220 were investigated using Kyoto Encyclopedia of Genes and Genomes (KEGG) enrichment analysis. Our data showed that the levels of TMEM220-AS1 and TMEM220 were reduced in HCC specimens and were associated with poor prognosis. TMEM220-AS1 suppressed the malignant behavior of tumor cells by targeting the TMEM220/β-catenin axis in HCC.

## Material and methods

### HCC patients and specimens

Eighty pairs of tumors and adjacent non-tumor tissues were collected from HCC patients who underwent R0 resection at the First Affiliated Hospital of Xi'an Jiaotong University between January 2012 and March 2014. Patients who were pathologically confirmed to have HCC and had complete clinical data were enrolled after providing written informed consent. Patients with HCC who had received preoperative treatment or had other tumors were excluded. The specimens were maintained at -80 °C. The study protocol (No: XJTU1AF2020LSK-123) was approved by the Ethics Committee of the First Affiliated Hospital of Xi'an Jiaotong University. Table 1 presents the clinical characteristics of the HCC patients.

### Cell culture and transfection

Human HCC cell lines (HCCLM3, Hep3B, HepG2, MHCC97H, and Huh7) were preserved in our lab 13. The normal hepatic cell line, MIHA, was obtained from Bnbio (Beijing, China) and cultured under standard conditions. TMEM220-AS1 and TMEM220 expression plasmids were generated by inserting cDNA into the pcDNA3.1 vector (V79020, Invitrogen, Carlsbad, CA, USA). TMEM220 shRNA (shTMEM220) was provided by GenePharma (Shanghai, China). The vectors were transfected into HCC cells using the Qiagen Effectene transfection reagent (301427, Valencia, CA, USA).

### RT-qPCR

RNA was extracted using TRIzol reagent (15596018, Invitrogen). TIANScript RT Kit (KR104, Tiangen Biotech, Beijing, China) was used for reverse transcription. Amplification was performed using the Bio-Rad CFX96 Touch Real-Time PCR Detection System (Bio-Rad, Hercules, CA, USA) and FS Universal SYBR Green Master kit (4913850001, Roche, Shanghai, China) following the manufacturer's instructions. The 2^-ΔΔCT^ method was used to analyze relative changes in gene expression. The primers used are listed in Supplementary Table 1.

### Western blotting (WB) analysis

Protein isolation was carried out using the RIPA Lysis Buffer System (sc-24948, Santa Cruz, CA, USA) and the concentration was measured using the Bradford protein assay kit (P0006, Beyotime, Shanghai, China). Then, 20 μg protein was subjected to SDS-PAGE (10%) and the bands transferred to PVDF membranes (IPVH00010, Millipore, Bedford, MA, USA). The membranes were incubated overnight at 4 °C with anti-TMEM220 (PA5-69312, Thermo Fisher Scientific, Waltham, MA, USA), anti-β-catenin (#8480, Cell Signaling Technology, Beverly, MA, USA), anti-β-actin (66009-1-Ig, Proteintech, Wuhan, China), and histone H3 (PTM-1001RM, PTM BIO, Hangzhou, China). The membranes were then incubated with HRP-bound secondary antibody (A0208 and A0216, Beyotime) and detected using an ECL system (WBLUF0500, Millipore). The resulting bands were scanned using an Amersham Imager 680 (GE Healthcare Life Sciences, Pittsburgh, PA, USA).

### Cell proliferation assay

For the cell counting kit-8 (CCK-8) assay, transfected HCC cells were suspended in complete DMEM and seeded into 96-well plates (2×10^3^ per well). The plates were supplemented with 10 μL CCK-8 solution (CK04, Dojindo Laboratories, Dojindo, Japan) per well and incubated for 3 h at 37 °C. The absorbance value (OD) was measured at 450 nm using a Multiskan FC microplate reader (Thermo Fisher Scientific). The EdU assay was conducted using the Cell-Light™ EdU Apollo®488 *In Vitro* Imaging Kit (C10310-1, RIBOBIO, Guangzhou, China) following the manufacturer's protocol, as previously described 7.

### Transwell assay

The migratory and invasive abilities of HCC cells were assessed using transwell chambers with or without Matrigel (356234, BD Biosciences, Franklin Lakes, NJ, USA). Cells (3×10^4^) in serum-free DMEM medium were seeded on the upper chamber and DMEM containing 10% FBS was added as a chemoattractant to the lower chamber. After 24 h incubation, 0.1% crystal violet was used to visualize the migrated or invaded HCC cells. Images of the membrane area were captured randomly and the cells counted.

### TCGA data analysis

TCGA data analysis was performed using the Gene Expression Profiling Interactive Analysis (GEPIA) webserver (http://gepia.cancer-pku.cn/) as previously described 14.

### Statistical analysis

Analysis of variance (ANOVA) with Tukey's multiple comparison test, Student's *t*-test, chi-squared test, and Mann-Whitney U-test were performed using GraphPad Prism version 8 (GraphPad Inc., San Diego, CA, USA). Survival in the two HCC subgroups was compared using the Kaplan-Meier method and log-rank test. Results from at least three independent repeats are shown as mean ± S.D. Statistical significance was set at* P*<0.05.

## Results

### The downregulation of TMEM220-AS1 is correlated with HCC patients' survival

**Figure 1:**
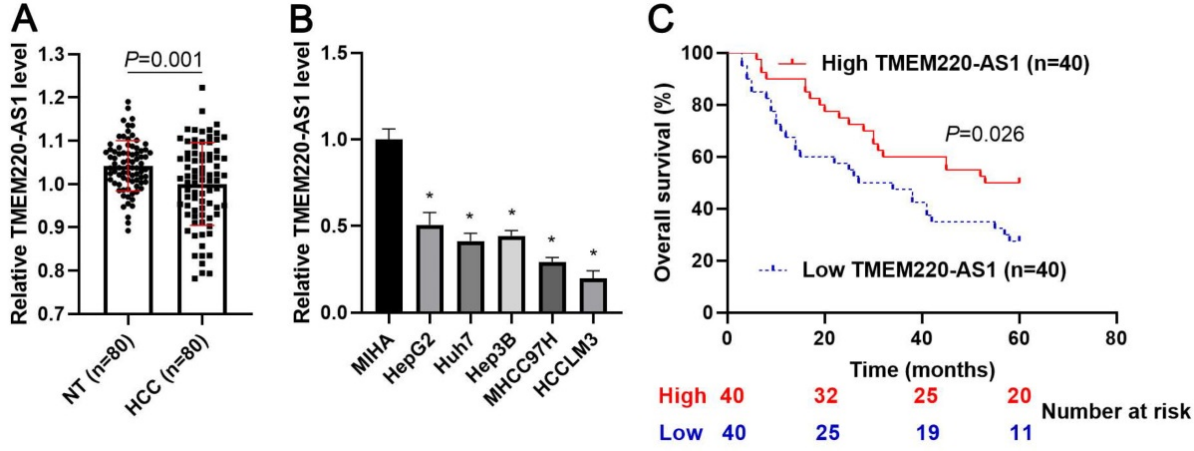
** Expression and clinical significance of TMEM220-AS1 in HCC. (A)** TMEM220-AS1 levels were detected in 80 pairs of HCC and nontumor (NT) tissues. **(B)** TMEM220-AS1 expression was assessed in MIHA, HCCLM3, Huh7, HepG2, Hep3B, and MHCC97H cells. **(C)** The survival of HCC patients with low versus high TMEM220-AS1 expression was compared. *P<0.05.

First, we demonstrated that TMEM220-AS1 expression was significantly reduced in tumor tissue samples compared with adjacent noncancerous tissues (*P*=0.001, Figure 1A). Consistent with our data, TCGA data also indicated that TMEM220-AS1 expression in HCC samples was markedly lower than that in normal tissues (*P*<0.0001, Supplementary Figure 1A). The expression of TMEM220-AS1 was downregulated in HCC cell lines, including HCCLM3, Huh7, Hep3B, HepG2, and MHCC97H, compared with MIHA cells (*P*<0.05, Figure 1B). The data in Table 1 show that low levels of TMEM220-AS1 were associated with malignant clinical parameters of HCC, including advanced TNM stage (*P*=0.014), venous infiltration (*P*=0.007), and tumor size > 5 cm (*P*=0.017). A close correlation between TMEM220-AS1 expression and the pathological stage of HCC was identified by analyzing the TCGA data (*P*<0.0001, Supplementary Figure 1B). Our follow-up results and TCGA data consistently suggested that the reduced TMEM220-AS1 levels correlated with poor prognosis in HCC patients (*P*<0.05, Figure 1C and Supplementary Figure 1C). Thus, our data indicates that TMEM220-AS1 is a potential predictive marker for HCC prognosis.

### TMEM220-AS1 suppresses the biological behavior of HCC cells

**Figure 2:**
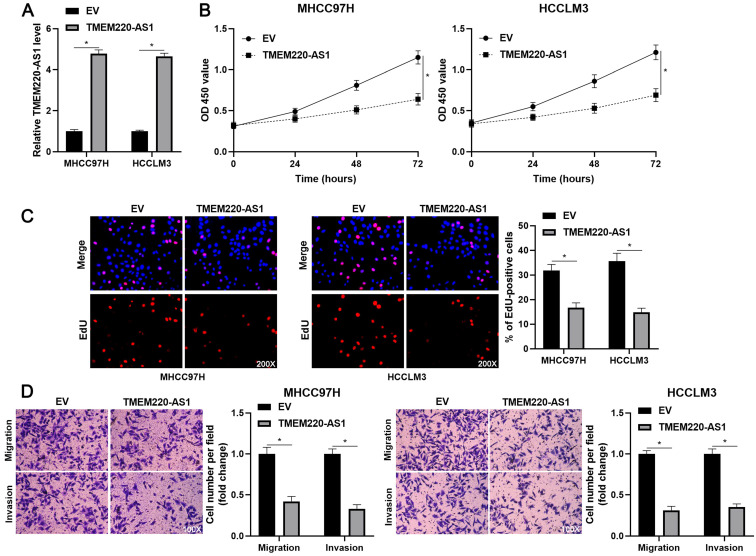
** TMEM220-AS1 inhibits HCC cell proliferation and mobility. (A)** MHCC97H and HCCLM3 cells were transfected with pcDNA3.1 vector carrying TMEM220-AS1 or with empty vector (EV) and then analyzed for TMEM220-AS1 expression using RT-qPCR. **(B)** TMEM220-AS1 overexpression suppressed HCC cell viability, as shown using CCK-8 assay. **(C)** EdU assay confirmed that TMEM220-AS1 overexpression decreased HCC cell proliferation. **(D)** The number of migrated and invaded HCC cells was reduced by TMEM220-AS1 overexpression. *P<0.05.

To determine the effects of TMEM220-AS1 on the malignant behavior of HCC cells, TMEM220-AS1 was ectopically expressed in MHCC97H and HCCLM3 cells (*P*<0.05, Figure 2A). The viability of HCC cells was significantly suppressed by TMEM220-AS1 overexpression (*P*<0.05, Figure 2B), as indicated by the CCK-8 assay. The cell proliferation assay confirmed that TMEM220-AS1 overexpression markedly reduced the percentage of EdU-positive HCC cells (*P*<0.05, Figure 2C). Subsequent transwell assays showed that TMEM220-AS1 overexpression reduced the number of migrated and invaded HCC cells (*P*<0.05, Figure 2D). Therefore, these results suggest that TMEM220-AS1 suppresses the proliferation and invasiveness of HCC cells.

### TMEM220 is upregulated by TMEM220-AS1 and downregulated in HCC

**Figure 3:**
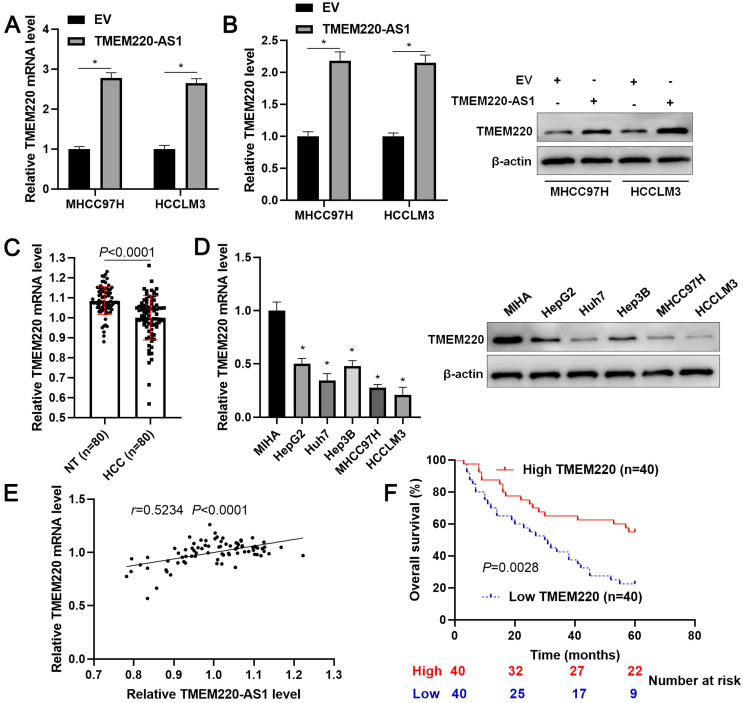
** TMEM220 is a target of TMEM220-AS1. (A)** HCC cells were transfected with pcDNA3.1 vector carrying TMEM220-AS1 or with empty vector (EV) and TMEM220 mRNA was detected using RT-qPCR. **(B)** Western blotting analysis revealed that TMEM-220-AS1 overexpression upregulated TMEM220 protein levels in HCC cells. **(C)** The levels of TMEM220 mRNA were measured in 80 pairs of HCC and nontumor (NT) tissues. **(D)** TMEM220-AS1 expression was assessed in MIHA, HCCLM3, Huh7, HepG2, Hep3B, and MHCC97H cells. **(E)** TMEM220-AS1 was positively associated with TMEM220 mRNA expression in HCC tissue samples. **(F)** The survival of HCC patients with low versus high TMEM220 expression was compared. *P<0.05.

TMEM220-AS1 is an antisense lncRNA of TMEM220. Thus, we elucidate the regulatory effect of TMEM220-AS1 on TMEM220 expression. As expected, we found that TMEM220-AS1 overexpression significantly increased TMEM220 mRNA and protein expression in MHCC97H and HCCLM3 cells (*P*<0.05, Figure 3A and 3B). However, the overexpression of TMEM220-AS1 did not affect the stability of TMEM220 mRNA in HCC cells (Supplementary Figure 2). On this basis, our clinical and TCGA data demonstrated that TMEM220 mRNA expression in HCC was significantly lower than in adjacent non-tumor tissues (*P*<0.0001, Figure 3C and Supplementary Figure 3A). TMEM220 levels were lower in HCC cell lines compared with MIHA cells (*P*<0.05, Figure 3D). Moreover, positive correlation between TMEM220 mRNA and TMEM220-AS1 expression was confirmed in HCC tissue samples (*P*<0.0001, Figure 3E and Supplementary Figure 3B). HCC patients with low TMEM220 expression had significantly shorter overall survival than patients with high TMEM220 expression (*P*=0.0028, Figure 3F). TCGA data also confirmed that low TMEM220 mRNA expression was associated with advanced pathological stage and poor HCC prognosis (*P*<0.01, Supplementary Figure 3C and 3D). Collectively, TMEM220 was a target for TMEM220-AS1, and its low expression was associated with poor clinical outcome in HCC.

### TMEM220 mediates the suppressive role of TMEM220-AS1 in HCC cells

**Figure 4:**
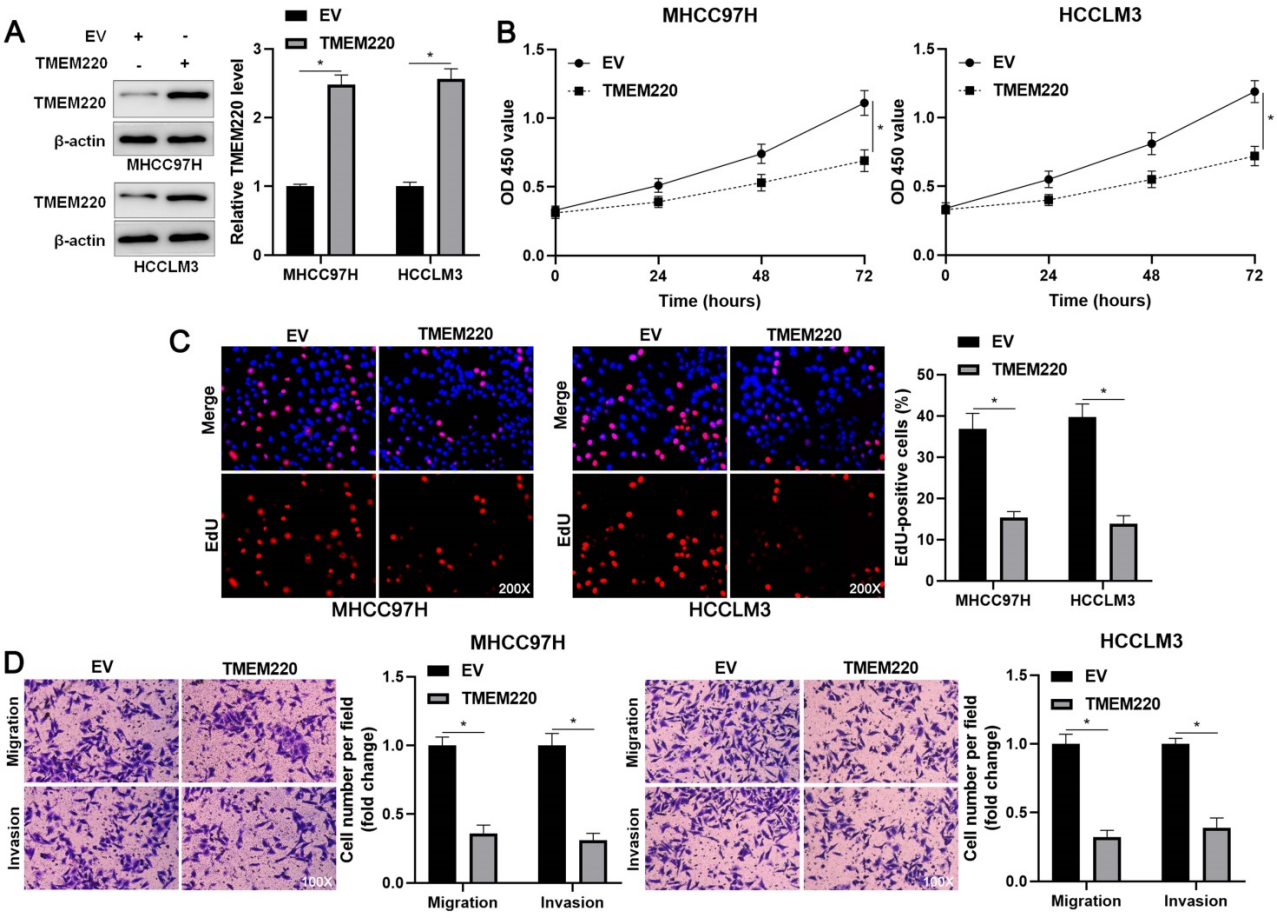
** TMEM220 suppresses the migration, invasion, and proliferation of HCC cells. (A)** MHCC97H and HCCLM3 cells were transfected with pcDNA3.1 vector carrying TMEM220 or with empty vector (EV) and TMEM220 analyzed using western blotting analysis. **(B)** TMEM220 overexpression suppressed HCC cell viability, as determined using CCK-8 assay. **(C)** EdU assay confirmed that TMEM220 overexpression weakened HCC cell proliferation. **(D)** The number of migrated and invaded HCC cells was decreased by TMEM220 overexpression. *P<0.05.

**Figure 5:**
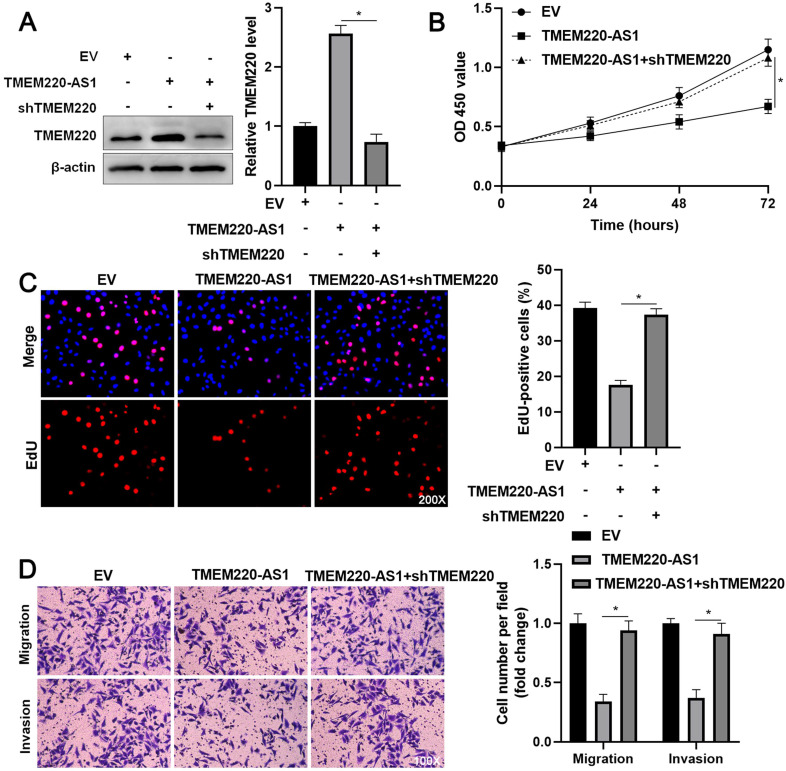
** TMEM220 knockdown reverses the inhibitory effect of TMEM220-AS1 in HCCLM3 cells. (A)** Western blot analysis showed that TMEM220 levels were reduced by TMEM220 shRNA (shTMEM220) in TMEM220-AS1-overexpressing HCCLM3 cells. (**B**) MTT, (**C**) EdU, and (**D**) transwell analyses demonstrated that TMEM220 knockdown reversed the suppressive role of TMEM220-AS1 in HCCLM3 cells. *P<0.05.

To investigate the regulatory role of TMEM220 in cell proliferation and mobility, we increased TMEM220 levels in MHCC97H and HCCLM3 cells (*P*<0.05, Figure 4A). TMEM220 overexpression significantly suppressed HCC cell proliferation, as indicated by the CCK-8 and EdU assays (*P*<0.05, Figure 4B and 4C). Furthermore, ectopic expression of TMEM220 suppressed the migratory and invasive abilities of HCC cells (*P*<0.05, Figure 4D). TMEM220 levels were downregulated in TMEM220-AS1-overexpressing HCCLM3 cells (*P*<0.05, Figure 5A). Notably, TMEM220 silencing reversed the inhibitory effect of TMEM220-AS1 in HCCLM cells (*P*<0.05, Figure 5B-D). Thus, TMEM220 is a functional effector of TMEM220-AS1 in HCC cells.

### TMEM220 regulates the Wnt/β-catenin pathway

**Figure 6:**
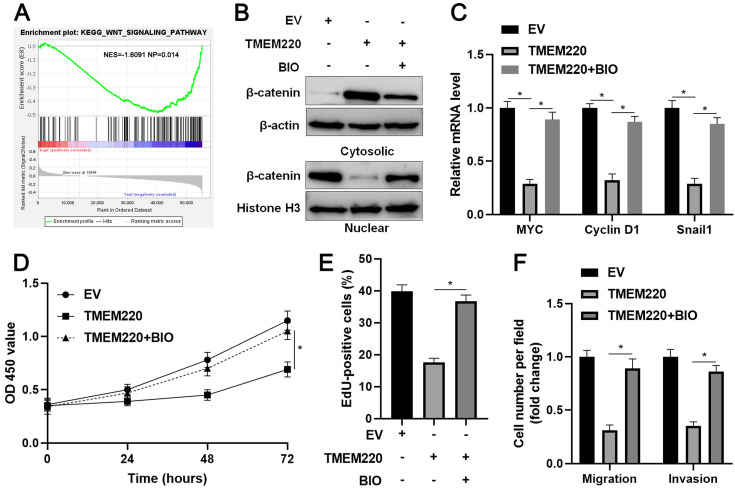
** TMEM220 regulates the Wnt/β-catenin pathway in HCC cells. (A)** KEGG enrichment analysis showed that the low TMEM220 level was correlated with the Wnt signaling pathway. **(B)** TMEM220-overexpressing HCCLM3 cells were treated with 2 μM of the GSK3β inhibitor, BIO, and western blotting was performed to detect β-catenin. **(C)** TMEM220-overexpressing HCCLM3 cells were treated with 2 μM of the GSK3β inhibitor, BIO, and RT-qPCR was performed to detect MYC, Cyclin D1, and Snail1 mRNA levels. (**D**) MTT, (**E**) EdU, and (**F**) transwell analyses demonstrated that BIO treatment reversed the suppressive role of TMEM220 in HCCLM3 cells. *P<0.05.

To explore the potential mechanism involved in the function of TMEM220 in HCC, KEGG enrichment analysis was conducted to explore the potential signaling pathways affected by TMEM220. We found that low TMEM220 expression was correlated with the Wnt signaling pathway (Figure 6A). Additionally, the subcellular distribution of β-catenin and the levels of its target genes were detected after TMEM220 overexpression in HCCLM3 cells. As expected, TMEM220 overexpression decreased the nuclear accumulation of β-catenin and reduced the mRNA levels of MYC, Cyclin D1, and Snail1 (*P*<0.05, Figure 6B and 6C). BIO, a GSK3β inhibitor, reversed TMEM220-induced Wnt/β-catenin pathway inactivation (*P*<0.05, Figure 6B and 6C). Moreover, BIO treatment eliminated the inhibitory role of TMEM220 in HCC cell proliferation and mobility (*P*<0.05, Figure 6D-F). Collectively, TMEM220 exerted a tumor-suppressive role in HCC by inactivating the Wnt/β-catenin pathway.

## Discussion

In this study, we first verified that TMEM220-AS1 is expressed at low levels in HCC cells. Previous studies have implicated epigenetic and transcriptional regulation in the dysregulation of lncRNAs in human cancers 15-17. For example, DNA hypermethylation leads to a loss of LINC00261 expression in lung cancer 18 and N6-methyladenosine (m6A) modification is responsible for the overexpression of lncRNA RP11 in colorectal cancer 19. Transcription factors such as p53, Myc, and HIFs, activate lncRNA expression 6, 20, 21. Thus, it is necessary to elucidate the mechanism underlying the downregulation of TMEM220-AS1 in HCC. LncRNAs are recognized as promising novel diagnostic and prognostic biomarkers for HCC 22, 23. Serum small extracellular vesicle (EV)-derived LINC00853 is significantly elevated and has been identified as a valuable diagnostic biomarker for early HCC 24. LINC00978 is highly expressed in the serum of patients with HCC compared with patients with hepatitis and cirrhosis as well as healthy controls 25. The overexpression of lncRNA H19 predicts poor clinical outcomes in patients with HCC 26. In this study, we revealed that reduced TMEM220-AS1 levels were closely correlated with advanced tumor stage, venous infiltration, tumor size >5 cm, and poor prognosis of HCC. Therefore, it is worth determining TMEM220-AS1 expression in the serum of HCC patients and analyzing its diagnostic value in future studies.

Functional experiments confirmed that TMEM220-AS1 overexpression inhibited the growth and mobility of HCC cells. As an antisense lncRNA of TMEM220, TMEM220-AS1 positively regulates TMEM220 expression in HCC. A previous study showed that AdipoQ AS lncRNA binds to AdipoQ mRNA and suppresses its translation 27. The lncRNA FOXC2-AS1 transcriptionally and post-transcriptionally enhances FOXC2 expression in osteosarcoma 28. TMEM220-AS1 overexpression consistently increased TMEM220 mRNA and protein levels in HCC cells. TMEM220 mRNA stability was not affected by TMEM220-AS1 overexpression in HCC cells. These results suggest that TMEM220-AS1 transcriptionally regulates TMEM220 expression in HCC cells.

TMEM205 and TMEM106C, two members of the TMEM family, are highly expressed in HCC 29, 30. TMEM205 is recognized as an independent prognostic marker and is correlated with the immune microenvironment in HCC 30. The upregulation of TMEM106C predicts poor HCC prognosis and enhances the malignancy of cancer cells 29. Conversely, our data showed that TMEM220 is downregulated in HCC. The reduced level of TMEM220 indicated reduced survival in patients with HCC. TMEM220 overexpression suppressed HCC cell migration, invasion, and proliferation, which was similar to the effects of TMEM220-AS1 on HCC cells. TMEM220 knockdown eliminated the role of TMEM220-AS1 in HCC cells. Collectively, TMEM220 is a tumor suppressor that mediates the anti-HCC effects of TEMEM220-AS1. KEGG enrichment analysis indicated that TMEM220 is inversely associated with the Wnt signaling pathway. TMEM220 overexpression significantly reduced the nuclear translocation of β-catenin and decreased the levels of MYC, cyclin D1, and Snail1 mRNA in HCC cells. The GSK3β inhibitor, BIO, a Wnt/β-catenin agonist, eliminated TMEM220-induced Wnt/β-catenin pathway inactivation. Several studies have demonstrated that the Wnt/β-catenin pathway plays a role in the occurrence and progression of HCC by enhancing target gene expression, including MYC, cyclin D1, and Snail1 31-33. We found that BIO treatment also enhanced the migration, invasion, and proliferation of TMEM220-overexpressing HCCLM3 cells. Thus, TMEM220 exerted a tumor-suppressive role in HCC by inactivating the Wnt/β-catenin pathway.

In summary, we demonstrated the downregulation of TMEM220-AS1 and TMEM220 in HCC and verified their potential in predicting poor prognosis. Both TMEM220-AS1 and TMEM220 inhibited the migration, invasion, and proliferation of HCC cells. TMEM220 mediated TMEM220-AS1's anti-HCC effects by inactivating the Wnt/β-catenin pathway. TMEM220-AS1 and TMEM220 may be promising prognostic biomarkers and therapeutic targets for HCC.

## Supplementary Material

Supplementary figures and table.

## Figures and Tables

**Table 1:** Correlation between TMEM220-AS1 expression and clinicopathologic characteristics in hepatocellular carcinoma

Characteristics	n=80	TMEM220-AS1 expression		*P*
		Low (n=40)	High (n=40)	
**Age (years)**				
<50	35	19	16	0.499
≥50	45	21	24	
**Sex**				
Male	63	32	31	0.785
Female	17	8	9	
**HBV infection**				
No	28	11	17	0.160
Yes	52	29	23	
**Serum AFP level (ng/mL)**				
<20	27	10	17	0.098
≥20	53	30	23	
**Tumor size (cm)**				
<5	26	8	18	0.017*
≥5	54	32	22	
**No. of tumor nodules**				
1	65	30	35	0.152
≥2	15	10	5	
**Cirrhosis**				
No	35	15	20	0.260
Yes	45	25	20	
**Venous infiltration**				
No	44	16	28	0.007*
Yes	36	24	12	
**Edmondson-Steiner grade**				
I+II	57	26	31	0.217
III+IV	23	14	9	
**TNM stage**				
I+II	63	27	36	0.014*
III+IV	17	13	4	

HBV, hepatitis B virus; AFP, alpha-fetoprotein; TNM, tumor-node-metastasis The “low” or “high” TMEM220-AS1 expression level was defined based on the cut-off value, which was defined as the median value of the cohort of patients tested. *Statistically significant.

## Abbreviations

IARC: International Agency for Research on Cancer
HCC: hepatocellular carcinoma
lncRNA: long noncoding RNA
TMEM220-AS1: transmembrane protein 220 antisense RNA 1
TCGA: The Cancer Genome Atlas
m6A: N6-methyladenosine
EV: extracellular vesicle
