# Supplementary material for: LncRNA TMEM220-AS1 suppresses hepatocellular carcinoma cell proliferation and invasion by regulating the TMEM220/β-catenin axis
Source: J Cancer. 2021 Sep 24;12(22):6805–13. doi: 10.7150/jca.63351 (PMC8517989; doi:10.7150/jca.63351)
Supplement: Supplementary file 1 — Supplementary figures and table. [file jcav12p6805s1.pdf]

Supplementary Table 1 The primer sequences used in this study

Gene	Primer	Sequence (5′-3′)
TMEM220-AS1	forward	CTTCACTCGCTGGCTGTAGG
	reverse	GGCCACACTTGCACCATATC
TMEM220	forward	CCTTGGTGCAGGTGGTGTA
	reverse	ACAGCCCACACCGTACAAAA
MYC	forward	TGGAACTTACAACACCCG
	reverse	CCTCGTCGCAGTAGAAAT
Cyclin D1	forward	CTGACACCAATCTCCTCAACG
	reverse	CTCACAGACCTCCAGCATCCA
Snail1	forward	CACACGCTGCCTTGTGTCT
	reverse	GGTCAGCAAAAGCACGGTT
β-actin	forward	CATGTACGTTGCTATCCAGGC
	reverse	CTCCTTAATGTCACGCACGAT

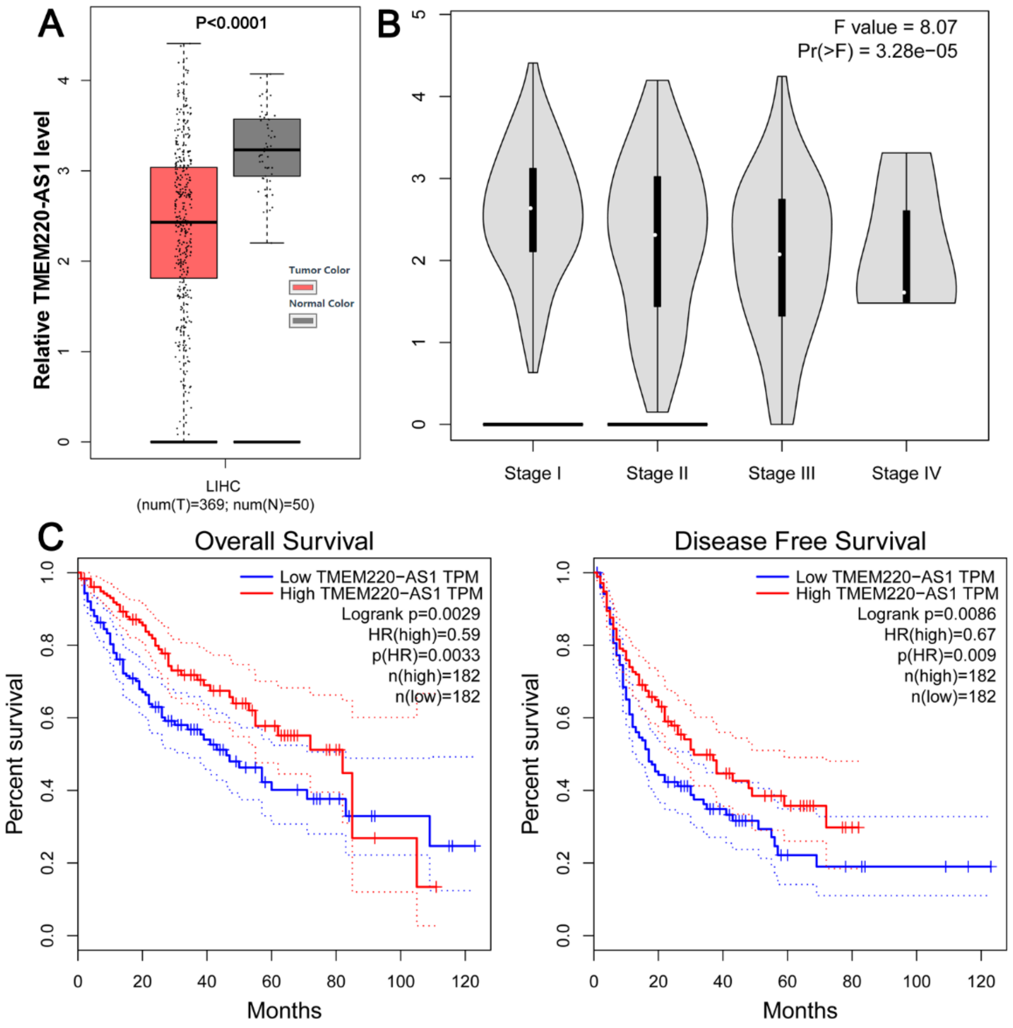

Supplementary Figure 1 The expression and clinical significance of TMEM220-AS1 in HCC based on TCGA data. (A) The expression difference of TMEM220-AS1 between HCC and normal liver tissues. (B) The correlation between TMEM220-AS1 expression and pathological stage of HCC. (C) The low expression of TMEM220-AS1 predicted poor prognosis of HCC.

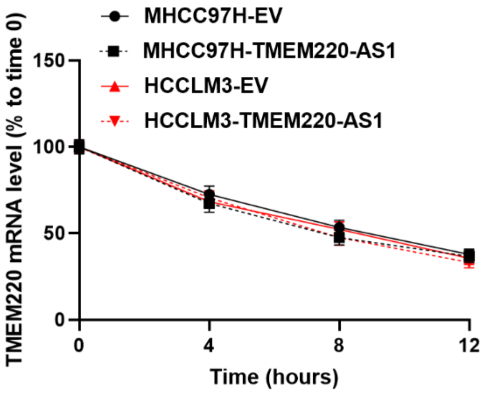

Supplementary Figure 2 TMEM220-AS1 overexpression dose not impact the stability of TMEM220 mRNA in HCC cells. MHCC97H and HCCLM3 cells that were transfected with pcDNA3.1 vector carrying TMEM220-AS1 or empty vector (EV) were treated with actinomycin D (5 μg/ml) to block transcription. qRT-PCR was performed to detect TMEM220 mRNA expression in different time points.

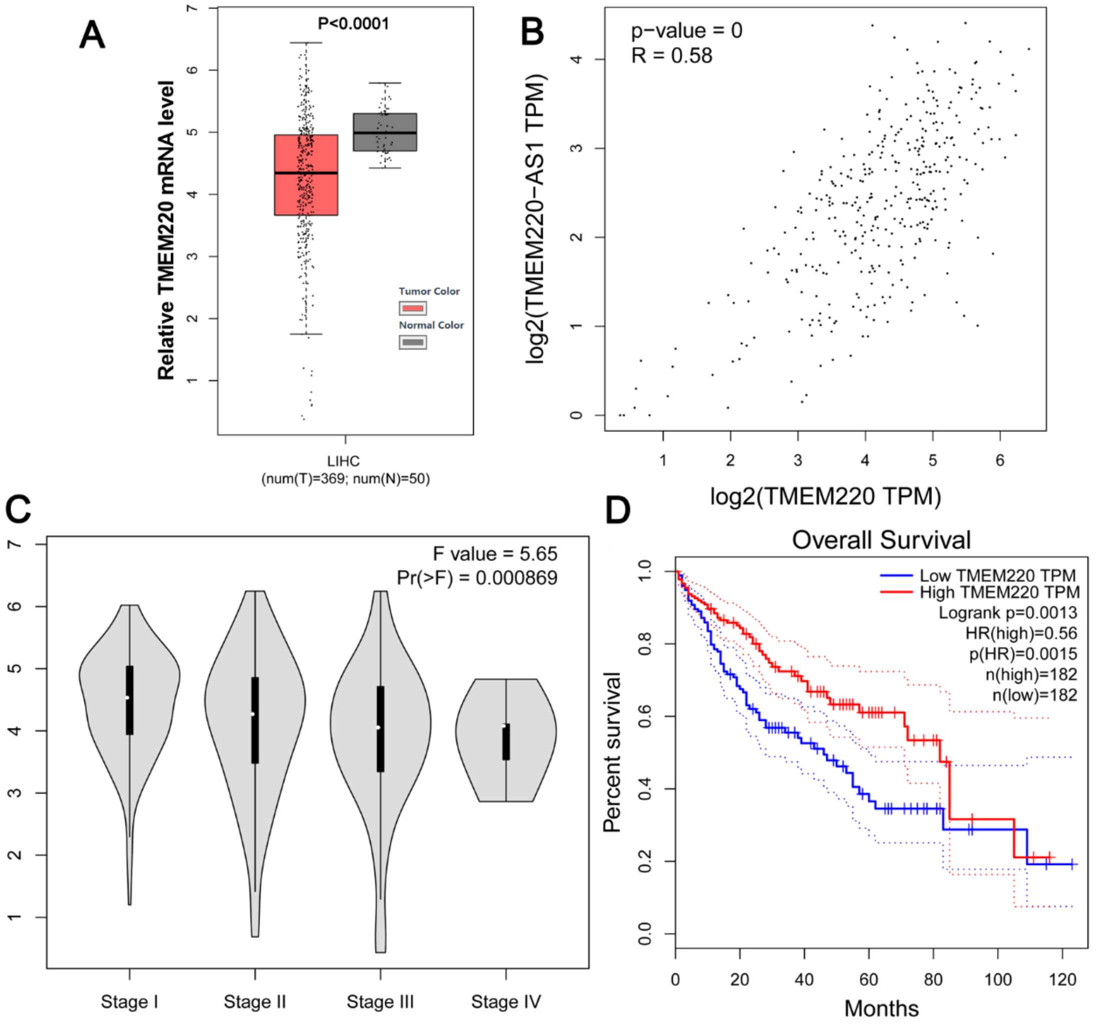

Supplementary Figure 3 The expression and clinical significance of TMEM220 in HCC based on TCGA data. (A) The expression difference of TMEM220 mRNA between HCC and normal liver tissues. (B) The correlation between TMEM220-AS1 and TMEM220 mRNA expression in HCC. (C) The correlation between TMEM220 mRNA expression and pathological stage of HCC. (D) The low expression of

TMEM220 predicted reduced overall survival of HCC.
